# Abundance of selected bacterial groups in healthy calves and calves developing diarrhea during the first week of life: Are there differences before the manifestation of clinical symptoms?

**DOI:** 10.3389/fmicb.2022.958080

**Published:** 2022-10-25

**Authors:** Karin Schwaiger, Julia Storch, Christoph Bauer, Johann Bauer

**Affiliations:** ^1^Unit of Food Hygiene and Technology, Institute for Food Safety, Food Technology and Veterinary Public Health, University of Veterinary Medicine, Vienna, Austria; ^2^Veterinary Office Landratsamt Fürstenfeldbruck, Fürstenfeldbruck, Germany; ^3^Department of Quality Assurance and Analytics, Bavarian State Research Center for Agriculture, Freising, Germany; ^4^TUM School of Life Sciences, Technical University of Munich, Freising, Germany

**Keywords:** diarrhea, microbiology, lactobacilli, dysbiosis, calves, newborn

## Abstract

**Background:**

Diarrhea is still the most common and economically most significant disease of newborn calves.

**Objective:**

Analysis of the development of selected bacterial groups in the feces of neonatal calves and its significance regarding diarrhea.

**Animals:**

A total of 150 newborn Simmental calves reared in 13 Bavarian farms were included in the study.

**Methods:**

Fecal samples of calves taken at 0/6/12/24/48/72/168 hours (h) since birth were analyzed qualitatively and quantitatively for aerobic and anaerobic bacteria, such as *Enterobacteriaceae, E. coli*, enterococci, and lactobacilli, using cultural, biochemical, and molecular-biological methods. Concurrently, the health status of the animals was recorded. The bacterial levels of healthy and diarrheic animals were compared using statistical methods. In addition, feces samples from calves that developed diarrhea were examined by ELISA for the presence of rotaviruses, coronaviruses, *E. coli* F5, and *Cryptosporidium (Cr.) parvum*.

**Results:**

Fifty-seven out of 150 calves (37.3 %) that were examined developed diarrhea within the first week of life. In the feces of calves with diarrhea on day 1 of life, the levels of aerobes, *Enterobacteriaceae*, and *E. coli* were significantly increased (*p* < 0.05), while no significant differences in enterococci and lactobacilli were found. In animals with the onset of diarrhea on day 2 after birth, the load of lactobacilli was significantly reduced up to 24 h before the manifestation of clinical symptoms compared to healthy calves. For enterococci, this was only the case on the day of the onset of diarrhea. In addition, the ratios of aerobic and anaerobic bacteria, *Enterobacteriaceae* or *E. coli* to lactobacilli, of calves with diarrhea starting on day 2 after birth are significantly higher than those of healthy calves. The detection frequency of specific pathogens in diarrheic calves increased over the first week of life.

**Conclusion:**

The results suggest that the incidence of neonatal diarrhea in calves is favored by low levels of lactobacilli in the feces. From this, the hypothesis can be derived that, in addition to an optimal supply of colostrum, the earliest possible administration of lactobacilli might reduce neonatal diarrhea in calves. However, this must be verified in a subsequent feeding experiment.

## Introduction

Diarrhea in newborn animals is a long-known health problem, possibly since humans started keeping domestic animals (Key et al., 2020). As early as 1793, Claß stated that “this evil happens frequently and many calves are lost” (Claß, 1793). Up to now, diarrhea is considered the most common and economically significant disease of young calves (Elze et al., 1994; Doll et al., 1995). A total of 5–10% of live-born calves die, and approximately half of all deaths among calves up to the first month of age are due to diarrhea (Kaske and Kunz, 2003; Brickell et al., 2009). The National Animal Health Monitoring System for U.S. dairy reported in 2014 that 56% of ill calves showed digestive disorders with a case fatality rate of 8.5%; most cases occurred in calves less than 1 month old (Urie et al., 2018).

Calf diarrhea is attributed to both non-infectious and infectious factors. Various enteric pathogens like bovine rotavirus (BRV), bovine coronavirus (BCoV), bovine viral diarrhea virus (BVDV), *Salmonella (S.) enterica, Escherichia (E.) coli, Clostridium (C.) perfringens*, and *Cryptosporidium (Cr.) parvum* along with newly emerging enteric pathogens such as bovine torovirus (BToV) and caliciviruses (bovine norovirus [BNoV] and Nebovirus) are known or considered to cause diarrhea (Cho and Yoon, 2014). In addition, factors such as housing type, colostrum intake, and hygienic conditions can be associated with field outbreaks and may favor the development of clinical symptoms and influence the severity and outcome of the disease (Lee et al., 2019).

Diarrhea is the most common clinical sign of gut dysbiosis and has been demonstrated in various animal species like dogs, cats, or horses (Guard et al., 2015; Suchodolski et al., 2015; Arroyo et al., 2020). Compared to healthy calves, feedlot cattle with hemorrhagic diarrhea showed significant increases in the relative abundance of *Clostridium, Blautia*, and *Escherichia*, and significant decreases in the relative abundance of *Flavobacterium, Oscillospira, Desulfonauticus, Ruminococcus, Thermodesulfovibrio*, and *Butyricimonas* (Zeineldin et al., 2018). Gomez et al. (2017) showed that the intestinal microbiota of healthy dairy calves appeared to be farm-specific, as were the changes during diarrhea. They suggested that dysbiosis can occur in diarrheic calves and is associated with changes in the predictive metagenomics function of the bacterial communities. In the present study, total aerobic and anaerobic colony forming units (cfu) in the fecal samples were determined to quantify the overall bacterial count development and the individual differences in the very first hours and days of life, as well as to see when the bacterial count plateau is normally reached. The selection of the further investigated bacterial groups was based on existing knowledge about the development of the microbiota according to which the intestinal tract of newborn calves is quickly colonized by *Enterobacteriaceae (E. coli*), clostridia, and enterococci. These bacteria are considered to predominate numerically during the first two days of the calves' lives. Furthermore, approximately 24 h after birth, lactobacilli establish themselves in the gastrointestinal tract and soon become a dominant species (Smith, 1965). Moreover, since lactobacilli are considered “beneficial microorganisms” (Pace et al., 2015), we wanted to investigate their development and impact on diarrhea in more detail.

It is not surprising that the fecal microbiota differs between healthy and diarrheic calves. However, hardly anything is known about the situation 1 or 2 days before clinical signs become manifest. Are there quantitative differences in selected bacterial groups in the feces of calves that stay healthy for the next few days versus those that develop diarrhea a day or two later? If that were the case, this might serve as a scientific basis for diarrhea prevention. To verify this hypothesis, we conducted a wide-ranging prospective study with newborn calves that combined clinical and extensive microbiological investigations during the first week of life.

## Materials and methods

### Animals

To have a representative number of diarrheic calves with a high probability of obtaining statistically sound results, 150 newborn animals (66 female and 84 male) of the Simmentaler breed were included in the study. To determine the number of animals, the incidence data from the studies by Girnus (2004) and Reski-Weide (2013) were used. The calculation was carried out using the software G^*^Power 3.1.9.7 (Faul et al., 2007). The calves were reared in 13 Bavarian farms (cows/farm: 55–165). Calf management and feeding designs were very similar during the first week of life. At all farms, the calves were placed in so-called calf-igloos or weather-protected individual boxes in the outdoor area within 24 h after birth. The time until the first colostrum (fresh, hand-milked) intake and the amount of colostrum ingested were recorded for each calf (see Supplementary Table). The calves were fed milk from the mother cow, milked by hand, 3–4 times per day for the first 5 days of life, after which the calves received a milk replacer. The cows were not treated with antibiotics during the perinatal period. Farmers were also instructed not to feed a starter containing probiotics to avoid data bias. In case of diarrhea, only oral electrolytes were administered, if necessary.

Daily clinical examination of all calves was performed for at least 8 days. Among the common clinical parameters, particular attention was given to sensorium, sucking reflex, skin turgor, eyeball position, rectal temperature, heart rate, respiratory rate, appetite, and feces characteristics (Stöber, 2012). Particular attention was paid to the consistency of the feces. For the diagnosis of “diarrhea”, the scoring system of the Clinic for Ruminants of the Veterinary Faculty of the University of Munich (Germany) was used (Prof. Wolfgang Klee, personal communication): feces that have the consistency of water or pea soup or that flow through spread fingers are classified as diarrhea.

Fecal samples were collected immediately after birth (0 h, meconium) and 6, 12, 24, 48, 72 (3 days), and 168 (7 days) h after birth for microbiological investigations. Animals showing diarrhea were excluded from data analysis from this point onward. Detailed data on the occurrence of diarrhea for each individual calf can be seen in Supplementary Table.

### Sampling

Feces samples were obtained directly by the farmers after being instructed by the authors of the present study. The anal region of the calves was cleaned (Bode Baktolin Waschlotion, Paul Hartmann AG, Heidenheim a. d. Brenz, Germany) and disinfected (Safe Sept Hautdesinfektion Pumpspray, Henry Schein, Berlin, Germany). Then, an industrial-clean glove was put on and the feces were removed with a gloved finger directly from the calf's anus. The glove was pulled inside out, knotted, and placed in a freezer bag. This procedure should ensure that contamination is largely avoided. The samples were stored at 4–8°C for a maximum of 48 h until further investigation.

### Microbiological examinations

The microbiological examinations were carried out as previously described (Schwaiger et al., 2020). Total aerobic and anaerobic cfu in the fecal samples were quantified by using the reference spatula method on Standard I Nutrient agar (Merck, Darmstadt, Germany) containing 7% defibrinated sheep blood (Fiebig, Idstein-Niederauroff, Germany) and Schaedler agar (Becton-Dickinson, Heidelberg, Germany) with 5% sheep blood and vitamin K (Fiebig, Idstein-Niederauroff, Germany; Merck, Darmstadt, Germany). This procedure ensures a detection limit of 10^2^ living bacteria/g feces (Gedek, 1974). The criteria for counting the different bacterial groups were growth on selective agar plates as well as morphology and color of the colonies. In addition, representative colonies were analyzed by microscopical, biochemical, and/or MALDI-TOF methods (see below).

The number of *Enterobacteriaceae* was determined using Gassner agar (Merck, Darmstadt, Germany), lactobacilli using LAMVAB-agar (Hartemink and Rombouts, 1999), and enterococci using citrate azide tween carbonate (CATC) agar (VWR, Darmstadt, Germany).

*Escherichia* (*E*.) *coli* were isolated on Gassner agar and biochemically confirmed by the production of β-D-glucuronidase and by the criterion of metabolizing sorbitol (Fluorocult agar, Merck, Darmstadt, Germany). Suspicious lactose-positive *Enterobacteriaceae* colonies not matching these criteria were investigated by API 20E (Biomerieux, Nürtingen, Germany). Lactose- and indole-negative strains were tested with antiserum *Salmonella* Omnivalent (Sifin, Berlin, Germany) for agglutination.

*Enterococcus* (*Ent*.) *faecalis* and *Ent. faecium* were isolated on CATC-Agar (citrate azide tween carbonate agar; Sifin, Berlin, Germany) and biochemically confirmed by testing their metabolism of xylose, mannitol, arabinose, and sodium-pyruvate (Bejuk et al., 2000).

For the identification of *Clostridium* spp., bacteria growing under anaerobic conditions on Schaedler-agar were sub-cultured under both aerobic and anaerobic conditions to exclude facultative anaerobic bacteria. The remaining obligatory anaerobes were identified at the genus level as *Clostridium* spp. using micro-morphological (Gram +/−, rods, spores) and biochemical criteria (oxidase −, indole +/−).

Bacteria of the genus *Lactobacillus* on LAMVAB-agar were identified at the genus level by microscopic (Gram +, rod, no formation of spores) and biochemical criteria (catalase−, oxidase−, indole +/−). The determination of the species was carried out by using MALDI-TOF-MS (Bruker Microflex™ LT; Bruker, Billerica, USA). Briefly, spectra from each isolate were obtained in accordance with the manufacturer's instructions using fresh and pure cultures. A small amount of culture material was transferred with a toothpick onto a 96-spot polished steel target plate and overlaid with one microliter of α-cyano-4-hydroxy-cinnamic acid. After drying at room temperature, species identification was performed using Bruker Microflex™ LT equipment (Bruker, Billerica, USA) and the Biotyper Real-Time Classification software v. 3.0 (Bruker Daltonics, Bremen, Germany).

In total, we examined 560 and 399 fecal samples from healthy and diarrheic calves, respectively, and more than 8,100 bacterial isolates were preserved for further identification.

In case of diarrhea, a digestive antigen sandwich enzyme-immunoassay (ELISA; BIO K 348, Bio-X Diagnostics, Rochefort, Belgium) was carried out according to the manufacturer's instructions to test for the presence of the most prevalent diarrhea pathogens BRV, BCoV, *E. coli* F5 (K99), and *Cr. parvum*.

### Statistical analysis

All statistical analyses were performed using the ‘R' software version 4.0.4 (http://www.r-project.org/). The cfu counts were log-transformed. The Wilcoxon rank-sum test was applied to compare the counts of bacterial groups of healthy and diarrheic calves. In addition, a Kolmogorov-Smirnov test was used to analyze the value distribution of the corresponding groups. The probability of the occurrence of diarrhea in the population of calves depending on the bacterial count at the species level was modeled by a logistic regression adjusting for the respective farm.

## Results

### General observations

Fifty-seven out of 150 calves (37.3 %) examined developed diarrhea within the first 7 days of life. The time of onset showed a bimodal distribution with peaks on days 2 and 6 (Table 1). Looking at the individual farms, the incidence of diarrhea was highly variable between 0 and 100% [in detail, from farm 1 to farm 13 (in %): 38/17/23/39/85/47/67/0/100/39/75/8, respectively]. For detailed information for each individual calf (see Supplementary Table).

**Table 1:** Detection of common pathogens in the feces of diarrheic calves (*n* = 57) using ELISA (BIO K 348) at different times after birth.

**Onset of diarrhea**		**Number of calves harboring pathogens**				
**Day**	**Number of animals**	**BRV**	**BCoV**	**Cr**	***E. coli* F5**	**BRV/Cr**
1	6	–	–	1	–	–
2	16	–	1	5	–	–
3	5	–	1	2	–	1
4	3	1	–	1	–	–
5	5	2	2	–	–	1
6	14	5	–	6	–	3
7	8	2	3	2	–	1

BRV, bovine rotavirus; BCoV, bovine coronavirus; Cr, Cryptosporidium parvum.

**Figure 1:**
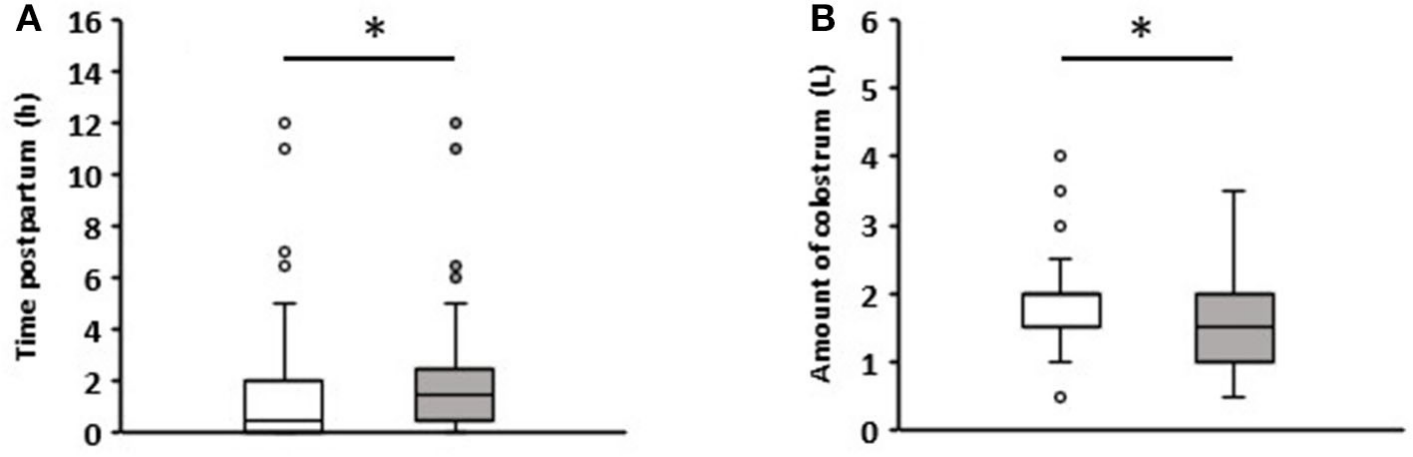
Comparison of colostrum intake data of newborn calves without (white boxes, *n* = 81) and with diarrhea (gray boxes, *n* = 57) in the first week of life. **(A)** Time to colostrum ingestion after birth. **(B)** Amount of colostrum ingested (Wilcoxon test, *p < 0.05).

The colostrum intake data are summarized in Figure 1. The colostrum was fed on average 1.7 h after birth. Calves that remained healthy in the first week received colostrum after an average of 1.5 h, while those that developed diarrhea ingesting it after 2 h on average (*p* = 0.04). All calves ingested an average of 1.78 (+0.72) liters (L) of colostrum. Calves that suffered from diarrhea during the first week consumed an average of 1.57 (+0.7) L, and those that remained healthy 1.92 (+0.7) L (*p* = 0.006). Detailed information on the time and volume of colostrum intake can be viewed in Supplementary Table for each individual calf.

### Pathogens in the feces of calves with diarrhea

The results of the ELISA assays are summarized in Table 1. *Cr. parvum* (*n* = 23) was detected most frequently, followed by BRV (*n* = 16) and BCoV (*n* = 7). *E. coli* F5 was not found in the samples analyzed. *Cr. parvum* antigen was already detectable in one sample on the first day of life, while BCoV and BRV were only found on the second and third day, respectively. In addition, it was apparent that later diarrhea occurs more frequently within the first week of life, the more often pathogens can be assigned to clinical events by ELISA. Detailed information about the pathogens detected for each individual calf with diarrhea can be seen in Supplementary Table.

### Quantity of selected bacterial groups in the feces of healthy and diarrheic calves

Based on the sampling design and the number of cases of diarrhea per day, the cases starting on days 1, 2, and 8 after birth were analyzed in more detail.

#### Diarrhea on day 1

**Figure 2:**
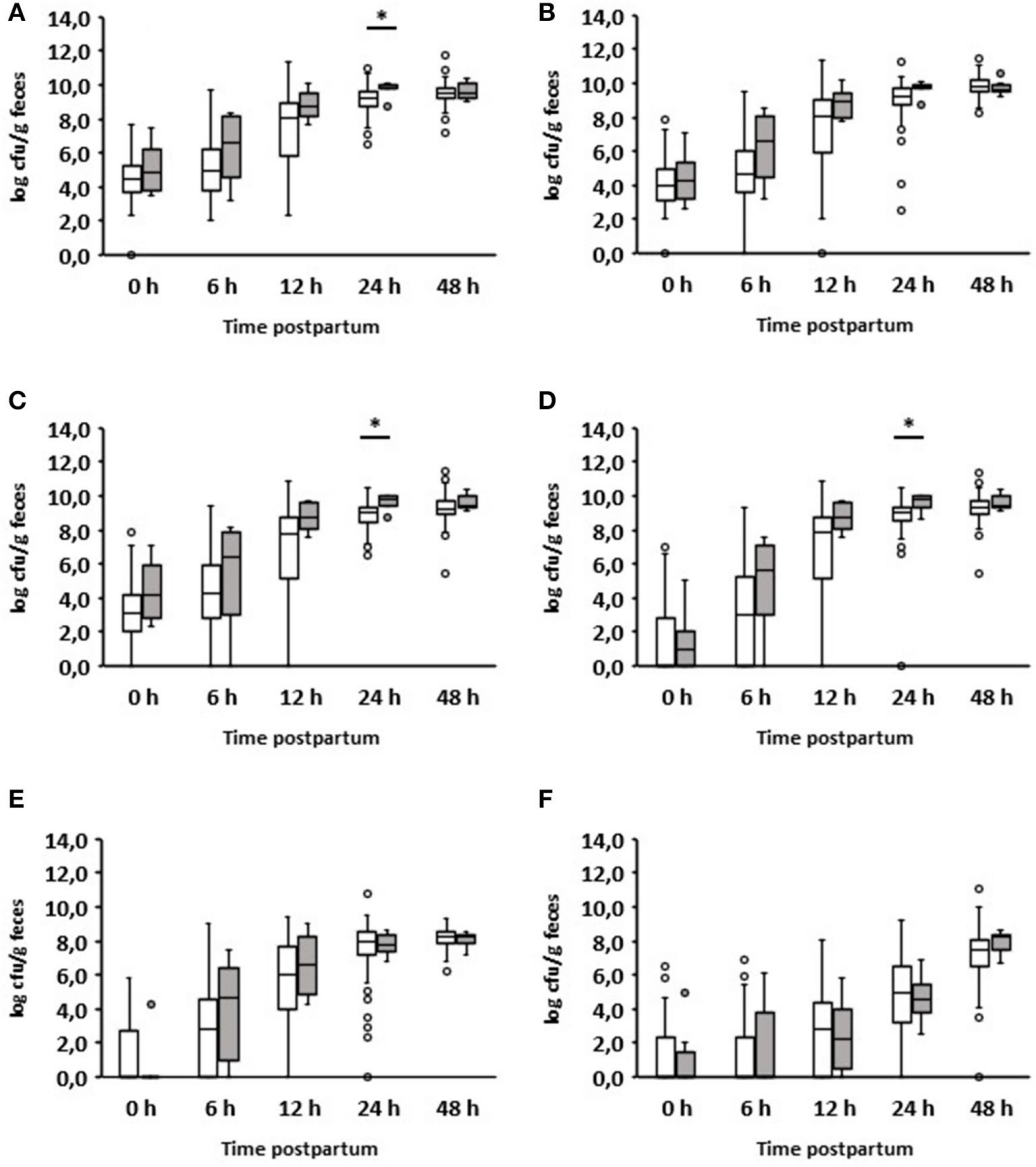
Fecal microbial contents of selected bacterial groups of healthy calves (white boxes, *n* = 144) and calves with diarrhea within 24 h after birth (gray boxes, *n* = 6). **(A)** Aerobic bacteria; **(B)** anaerobic bacteria; **(C)** *Enterobacteriaceae*; **(D)** *E. coli*; **(E)** *Enterococcus* spp.; **(F)** *Lactobacillus* spp. (Wilcoxon test, *p < 0.05).

Six out of 150 calves suffered from diarrhea during the first 24 h after birth (Table 1). The quantitative analysis showed a significant increase in aerobic bacteria (*p* = 0.029), *Enterobacteriaceae* (*p* = 0.013), and *E. coli* (*p* = 0.019) in the feces of diarrheic calves (Figure 2). Interestingly, these results were already apparent 12 h after birth. No significant differences could be seen between healthy and diarrheic calves regarding anaerobic bacteria, lactobacilli, and enterococci. On day 2 of life, the counts of the bacterial groups analyzed did not differ between healthy and diarrheic calves. On average, both groups ingested almost the same amount of colostrum at the first feeding (healthy calves: 1.79 L; diarrheic calves: 1.6 L).

#### Diarrhea on day 2

**Figure 3:**
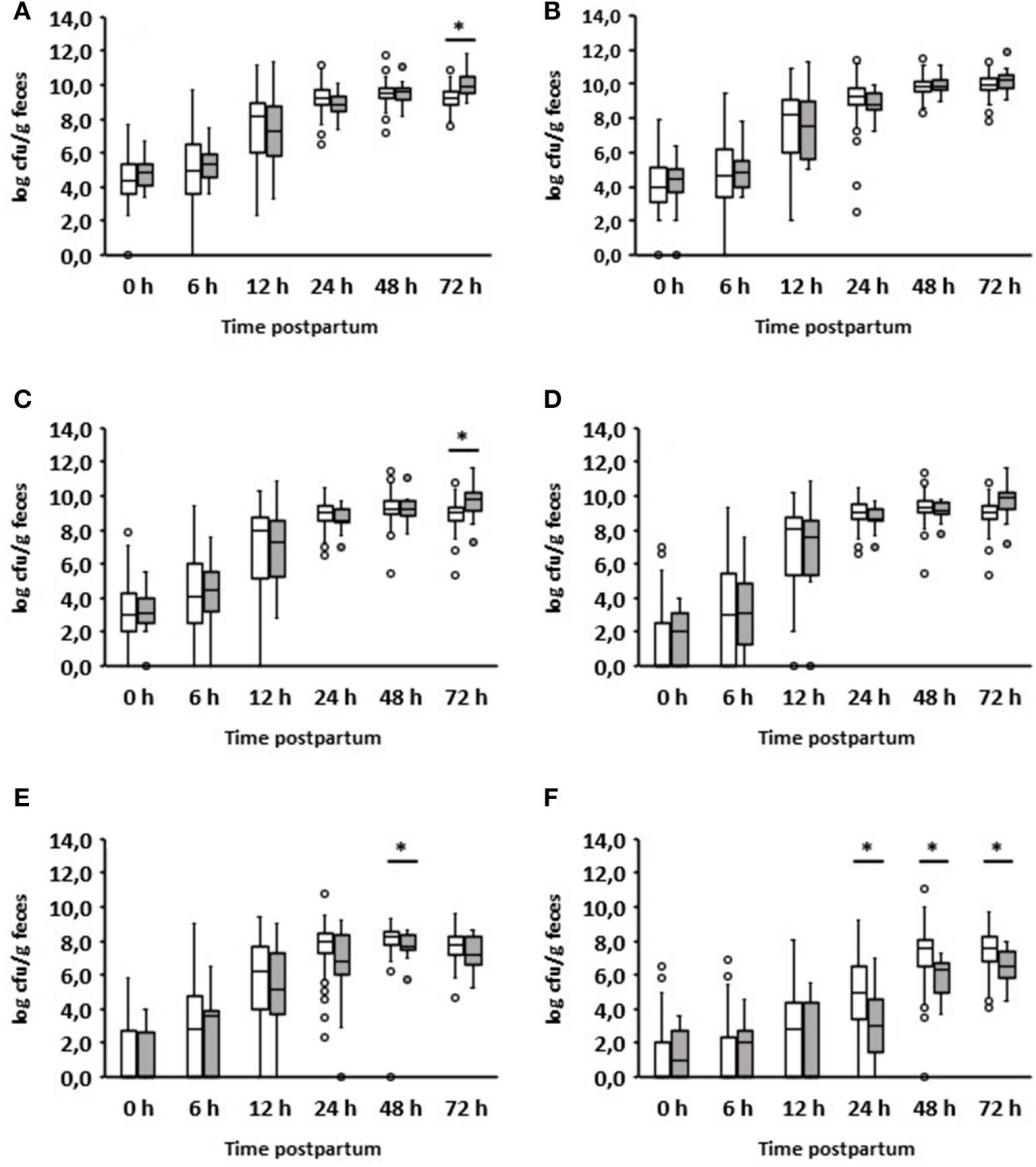
Development of the fecal microbial contents of selected bacterial groups during the first 72 h after birth. **(A)** Aerobic bacteria, **(B)** anaerobic bacteria, **(C)** *Enterobacteriaceae*, **(D)** *E. coli*, **(E)** *Enterococcus* spp., **(F)** *Lactobacillus* spp.; white boxes: values of healthy calves (*n* = 134–128); gray boxes: values of calves with diarrhea onset on day 2 (*n* = 16) (Wilcoxon test, *p < 0.05).

As already mentioned, most diarrheal diseases (*n* = 16) occurred on day 2 after birth (Table 1). Interestingly, the group of calves with diarrhea on day 2 consumed significantly less colostrum on average than those that remained healthy (1.33 vs. 1.86 L; *p* = 0.0025). The results of the bacteriological investigation are shown in detail in Figure 3. The development of fecal bacterial counts of aerobes, anaerobes, *Enterobacteriaceae*, and *E. coli* was almost the same during the first 48 h after birth in both calves with and without diarrhea on day 2. However, 1 day after the onset of diarrhea, diarrheic calves had significantly increased levels of aerobic bacteria (*p* = 0.000097) and *Enterobacteriaceae* (*p* = 0.002557; Figures 3A,C). While a similar trend was observed for *E. coli* (*p* = 0.0013; Figure 3D), this was not the case for anaerobic bacteria.

When evaluating the development of lactobacilli, it was found that these counts were significantly reduced 24 h before the manifestation of diarrhea compared to calves that remained healthy (*p* = 0.01). This was also the case on days 2 and 3 after birth. With regard to the number of enterococci, a significant difference could only be found in the 48-h samples. It should be noted that, first, a growth reduction in enterococci and lactobacilli was observed, and then an increase in aerobic counts, *Enterobacteriaceae*, and *E. coli*.

**Figure 4:**
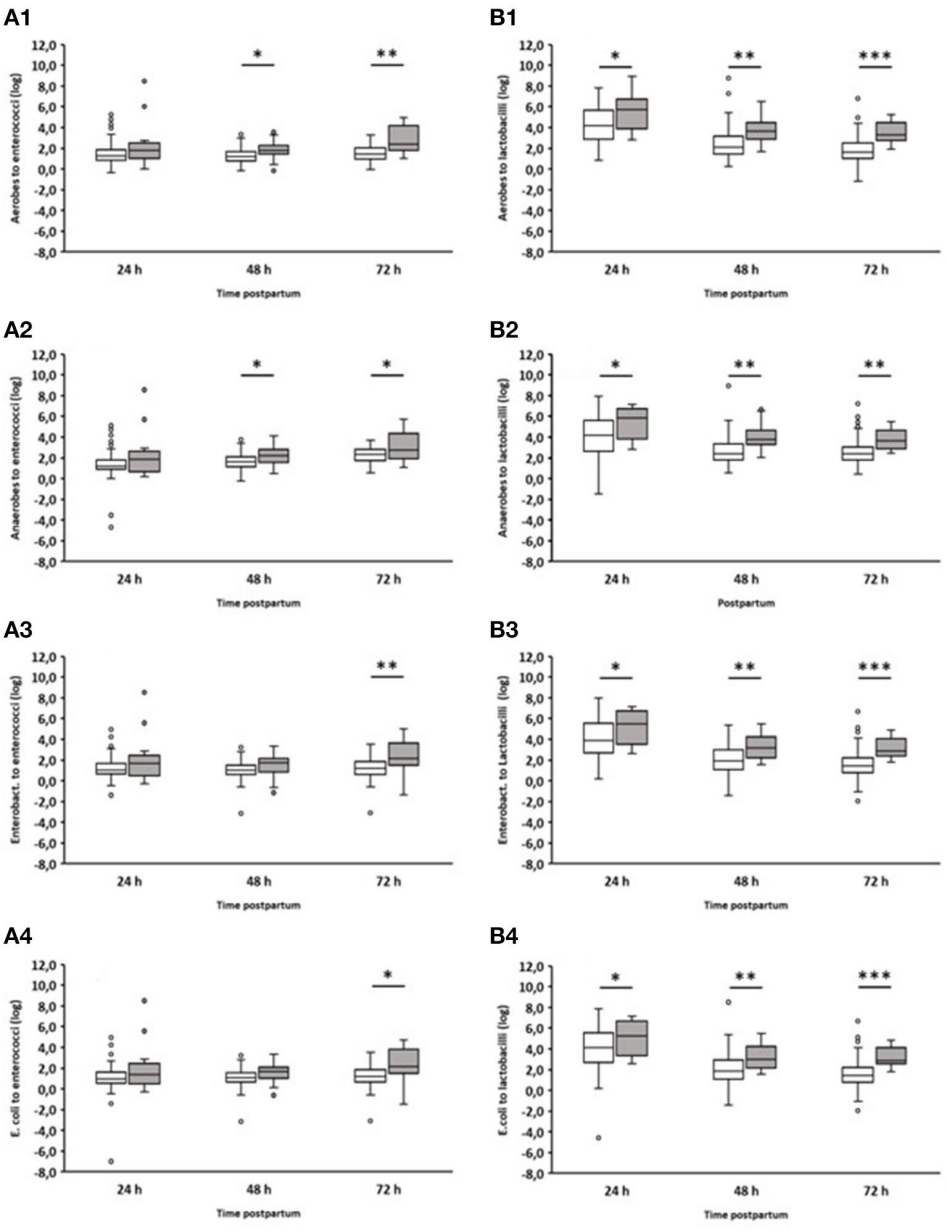
Ratio of counts (cfu/g feces) of specific bacterial groups to *Enterococcus* spp. or *Lactobacillus* spp. during the first 72 h after birth. **(A1)** Aerobic bacteria to enterococci; **(A2)** anaerobic bacteria to enterococci; **(A3)** *Enterobacteriaceae* to enterococci; **(A4)** *E. coli* to enterococci; **(B1)** Aerobic bacteria to lactobacilli; **(B2)** anaerobic bacteria to lactobacilli; **(B3)** *Enterobacteriaceae* to lactobacilli; **(B4)** *E. coli* to lactobacilli. White boxes: values of healthy calves (*n* = 127–133); gray boxes: values of calves with diarrhea onset on day 2 (*n* = 16; Wilcoxon test, *p < 0.05, **p < 0.001, ***p < 0.00001).

In addition, we analyzed the ratio of fecal aerobic counts, anaerobic counts, *Enterobacteriaceae*, and *E. coli* in comparison to enterococci and lactobacilli of healthy and diarrheic calves during the first three days of life (Figure 4). In the case of lactobacilli, it was noticeable that the ratios obtained for animals suffering from diarrhea on day 2 were significantly higher than those obtained for healthy calves. This was evident as early as 24 h before the clinical manifestation of diarrhea. Similar results were also found for enterococci, albeit to a much lesser extent.

**Figure 5:**
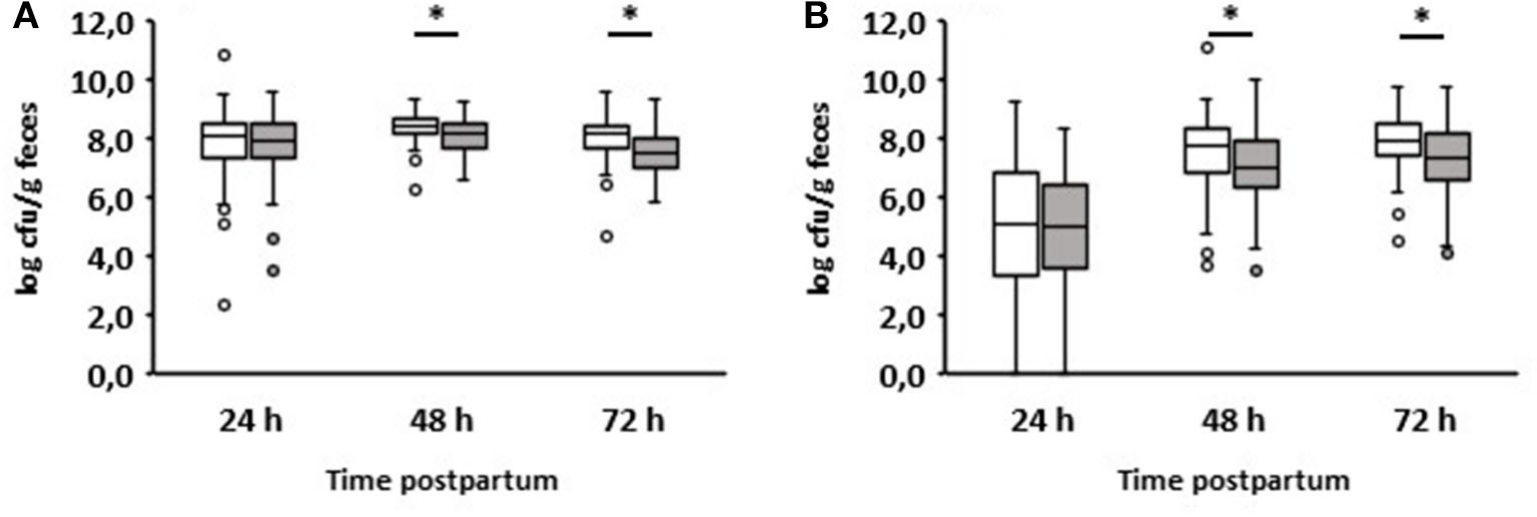
Counts of enterococci **(A)** and lactobacilli **(B)** in calves‘ feces. White boxes: feces of healthy calves from farms where no diarrhea occurred on day 2 of life (*n* = 49). Gray boxes: feces of healthy calves from farms where diarrhea occurred on day 2 of life (*n* = 79; Wilcoxon test, *p < 0.05).

**Figure 6:**
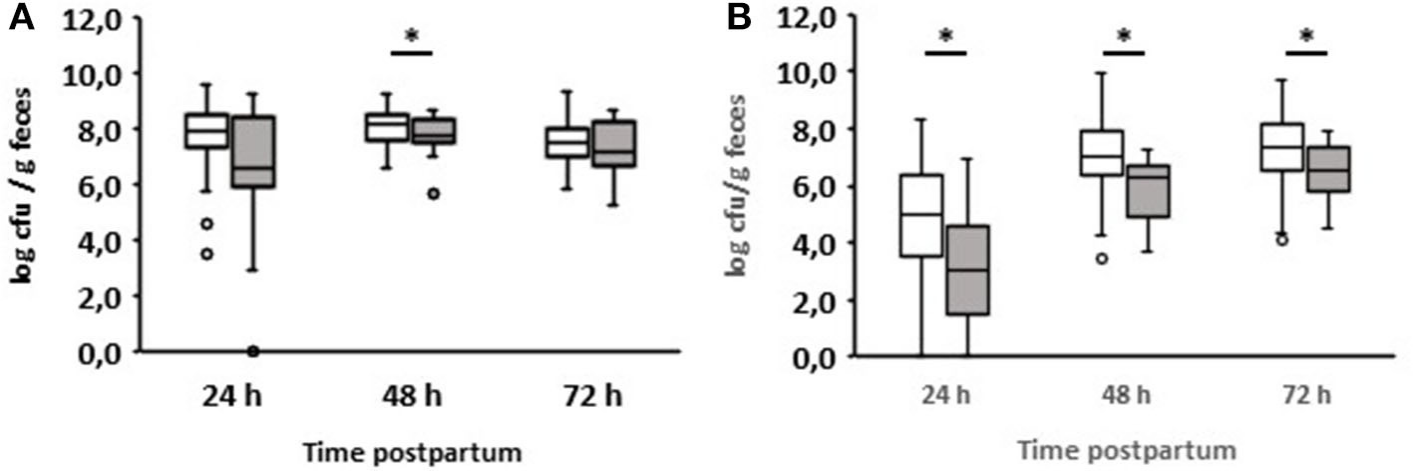
Counts of enterococci **(A)** and lactobacilli **(B)** in feces of calves from farms where diarrhea occurred on day 2 of life. White boxes: feces of healthy calves (*n* = 79). Gray boxes: feces of calves with diarrhea onset on day 2 of life (*n* = 16; Wilcoxon test, *p < 0.05).

We also compared enterococci and lactobacilli of healthy calves from farms with diarrhea with the counts of calves from farms without diarrhea on day 2 during the first three days of life (Figure 5). As a result, the numbers of enterococci and lactobacilli were significantly reduced on days 2 and 3 in healthy calves from farms with diarrhea on day 2, although the numbers of enterococci and lactobacilli were almost the same in both groups 24 h after birth. When comparing the numbers of fecal enterococci and lactobacilli of healthy and diarrheic calves from farms with diarrheic calves on day 2, a similar result as shown in Figures 3E,F emerged: bacteria of the genus *Lactobacillus* was significantly reduced in the feces of calves with diarrhea (Figure 6). There was no correlation between the volume of colostrum consumption or the first feeding time and the concentration of lactobacilli in the feces (data not shown).

#### Diarrhea From day 3 to day 7

Only five calves developed diarrhea 3 days after birth (Table 1). No differences in the levels of the selected bacterial groups could be detected between healthy and diarrheic calves. Since no feces samples were collected between days 4 and 6, a detailed evaluation during this period is not possible. The colostrum intake of the diarrheic and healthy calves during this period did not differ significantly (*p* = 0.112). However, it is notable that 14 cases of diarrhea occurred on day 6 after birth when BRV and/or Cr could be identified (Table 1).

#### Diarrhea on day 8

**Figure 7:**
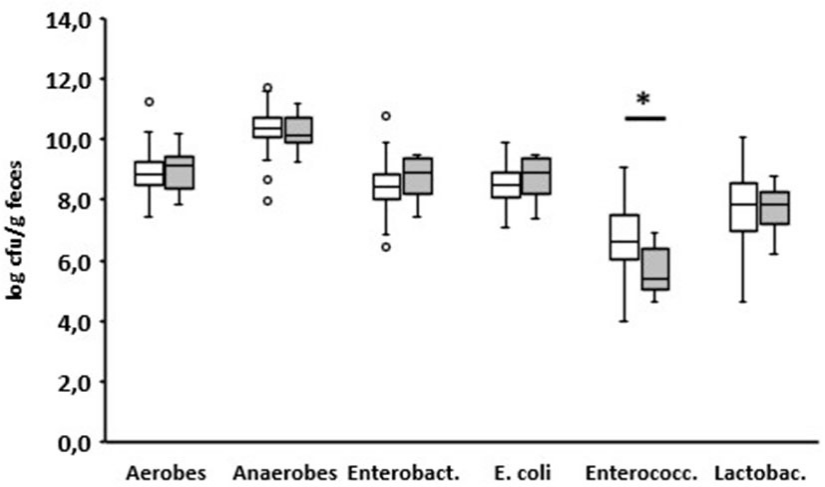
Fecal microbial contents of selected bacterial groups of healthy calves on day 7 after birth. White boxes: values of calves that remain healthy; gray boxes (*n* = 101): values of calves with diarrhea starting the next day (*n* = 8) (Enterobact., *Enterobacteriaceae*; Enterococc., *Enterococcus* spp.; Lactobac., *Lactobacillus* spp.; Wilcoxon test, *p < 0.05).

On day 8 after birth, eight of the 150 examined calves developed diarrhea (Supplementary Table). When fed for the first time, these eight calves ingested almost the same amount of colostrum as the animals that had remained healthy until that point (1.85 vs. 1.92 L, *p* = 0.8962). One day before, none of the numbers of aerobic bacteria, anaerobic bacteria*, Enterobacteriaceae, E. coli*, and lactobacilli changed in the feces in comparison to the fecal samples of animals that remained healthy. Only the number of enterococci was significantly reduced in the animals suffering from diarrhea the next day (*p* < 0.05; Figure 7).

Thirty-eight different species of Lactobacilli were detected in the feces of all calves during the whole investigation period. The most common species were identified as *L. brevis, L. fermentum, L. mucosae, L. murinus, L. parabuchneri, L. paracasei, L. plantarum, L. reuteri, L. rhamnosus*, and *L. salivarius*. The logit model showed that, with increasing concentration of *L. reuteri*, the probability of occurrence of diarrhea decreased; variable “a” was significant with *p* = 0.036.

## Discussion

Diarrhea in newborn calves is a multifactorial disease, and a variety of infectious and non-infectious criteria play a role in its development. In our study, about one-third of newborn calves showed diarrhea during the first 8 days of life. This incidence rate is comparable to that of previous investigations, which were conducted in the same geographic area and with the same cattle breed (Girnus, 2004; Reski-Weide, 2013). It is noteworthy that analysis of the frequency of diarrhea cases per day shows two peaks (day 2 and day 6 after birth), an observation that was already described by Bendali et al. (1999).

Not surprisingly, calf colostrum supply has a significant impact on diarrhea incidence—the earlier the calves were supplied with colostrum, and the higher the amount ingested, the lower the incidence of diarrhea. This fact has been repeatedly confirmed in numerous publications (Fallon and Harte, 1983; Donovan et al., 1998).

For reasons already discussed in our previous study (Schwaiger et al., 2020), we decided to apply cultivation techniques. Admittedly, it is known that a large proportion of intestinal bacteria cannot be detected using conventional bacteriology (Zoetendal and Mackie, 2005). However, these perhaps seemingly old-fashioned methods were better suited for the purposes of the present study than next-generation sequencing (NGS) techniques because they allow absolute quantification of cell numbers instead of relative abundances. Additionally, all cultivated isolates can be differentiated down to species level, whereas the composition of the microbiota determined by NGS is characterized mostly at the phylum or genus level. To gain reliable new insights into the kinetics of the (cultivable) microbiota of newborn healthy and diarrheic calves down to the species level, cultural techniques were combined with MALDI-TOF.

Calves that developed diarrhea within the first day of life had significantly higher concentrations of aerobic bacteria, anaerobic bacteria, *Enterobacteriaceae*, and *E. coli* in the feces. The high levels of these bacterial groups may be related to poor hygiene during or immediately after birth. For example, Klein-Jöbstl et al. (2014) demonstrated that cleaning the calving area after each calving significantly reduces the odds of calf diarrhea on farms. Apart from *Cr. parvum*, we were unable to detect a specific diarrhea-associated pathogen in the feces of this group of calves by ELISA. Therefore, it is conceivable that a high bacterial load may have disturbed the intestinal microbiota, which is still very unstable at that time, resulting in diarrhea.

As already mentioned, we detected *Cr. parvum* antigen in the feces of one calf with the onset of diarrhea on day 1. Since the development cycle of *Cr. parvum* takes about 3–6 days (Rommel, 2000), it is rather unlikely that the diarrhea of this calf was caused by *Cr. parvum*. Instead, it seems more likely that the detected particles of *Cr. parvum* have passed through the gastrointestinal tract without going through a stage of development. This must also be considered about the occurrence of *Cr. parvum* on day 2 of life. Since this single-celled organism is transmitted by fecal contamination, the positive ELISA result might be used as an indicator of poor hygiene during the perinatal phase.

Evaluation of the data of calves that developed diarrhea on the second day after birth shows that 24 h before the onset of diarrhea the number of lactobacilli was significantly reduced compared to healthy calves. This fact was also found both on the day on which diarrhea began and on the following day. Enterococci were significantly reduced only on the day diarrhea began. Interestingly, an increase in the numbers of aerobic bacteria, *Enterobacteriaceae*, and *E. coli* could only be observed one day after the onset of diarrhea.

The ratio of different bacterial taxa, especially Firmicutes to Bacteroidetes, is a frequently used parameter to describe gastrointestinal dysbiosis (Youmans et al., 2015; Bin et al., 2018). In fact, we could see a clear shift in the ratios of the numbers of aerobic bacteria, anaerobic bacteria, *Enterobacteriaceae*, and *E. coli* to lactobacilli. In the case of enterococci, this shift in the ratios was less pronounced (Figure 4). It is noteworthy that significant differences in the ratio of anaerobic bacteria to lactobacilli or enterococci were found between healthy calves and calves suffering from diarrhea, while this was not the case when comparing only the anaerobic bacterial counts (Figure 3). These results indicate that the proportions of aerobic bacteria, anaerobic bacteria, *Enterobacteriaceae*, and *E. coli* to lactobacilli have shifted to the disadvantage of the latter. The same is probably the case for enterococci, albeit to a lesser extent.

The reason why lactobacilli in calves that develop diarrhea do not increase as much as in animals that remain healthy cannot be clarified without further elaboration. As mentioned above, we were able to show the influence of colostrum supply on the incidence of diarrhea. However, there was no correlation between the amount of colostrum consumed or the first feeding time and the concentration of lactobacilli in the feces (data not shown). This agrees with the results of Fischer et al. (2018), who only found a relative reduction of lactobacilli in the colon when colostrum intake was delayed by 12 h. Since the components of the colostrum were not analyzed, we cannot make any statement about possible differences in the composition and the growth of lactobacilli.

An interesting aspect can be shown by comparing the fecal counts of enterococci and lactobacilli of healthy calves from farms with diarrhea with calves from farms without diarrhea on day 2 during the first 3 days of life (Figure 5): the numbers of enterococci and lactobacilli were significantly reduced on days 2 and 3 in healthy calves from farms with diarrhea on day 2, although the numbers of enterococci and lactobacilli were almost the same in both groups 24 h after birth. This discrepancy can result from different farm management systems. A so-called “farm effect” on the composition of the fecal microbiota has been observed by Weese and Jelinski (2017). However, as already mentioned, no major differences regarding the handling of newborn calves could be found. In addition, when comparing healthy and diarrheic calves from farms with diarrheic calves on day 2, bacteria of the genus *Lactobacillus* (and, to a lesser extent, also of the genus *Enterococcus*) were significantly reduced in the feces of calves with diarrhea (Figure 6). Recently, we showed that fecal samples from twin calves revealed higher similarity in single-strand conformation polymorphism profiles compared to their coresidents, indicating that the individual microbiota might be genetically or epigenetically influenced (Mayer et al., 2012).

The gut microbiota-modulating effects of enterococci and especially lactobacilli are discussed and demonstrated (Azad et al., 2018; Shin et al., 2019). It is well known that bacteria of both genera produce various compounds that inhibit the growth of bacteria. For example, enterocins were detected in cultures of *Ent. faecium, Ent. faecalis, Ent. Hirae*, and *Ent. durans* (Hernández-González et al., 2021), while strains of *L. reuteri* produce reuterin and reuterocycline (Talarico et al., 1988; Gänzle, 2004). In addition, growth-inhibition substances like organic acids, hydrogen peroxide, diacetyl, and ethanol are synthesized by lactobacilli (Millette et al., 2007; Vieco-Saiz et al., 2019). Because of this metabolic performance, and because of the competition for binding sites on the intestinal mucosa, enterococci and lactobacilli prevent the proliferation of bacteria and appear to be important for the development and stability of a eubiotic microbiota (Mokoena, 2017). The consequence of a decrease in enterococci and lactobacilli is an increase in the numbers of aerobic bacteria, *Enterobacteriaceae*, and *E. coli*.

*L. reuteri*, which is one of the *Lactobacillus* species predominant in calves (Schwaiger et al., 2020), seems to play a special role regarding their intestinal health. Delayed development of this species during the first 3 days of life favors the likelihood of diarrhea in the first week of life. The beneficial properties of *L. reuteri* are frequently discussed. In addition to the antibiotic effects mentioned above, inhibitory effects on viruses (especially rotavirus) as well as cryptosporidia are described (Alak et al., 1997; Glass et al., 2004; Seo et al., 2010; Ang et al., 2016). Our results contradict those of the recently published investigation by Slanzon et al. (2022). According to this study, *L. reuteri* occurs more frequently in the fecal microbiota of calves with gastrointestinal diseases. However, it should be noted that (1) calves of other breeds (Holstein, Jersey, Jersey-cross, and beef-cross vs. Simmentaler) and (2) a different age group (4–21 days vs. 1–7 days) were examined.

Looking at the counts of the selected bacterial groups on day 7 (Figure 5), a significant difference between calves that remained healthy and calves suffering from diarrhea the next day could only be found for enterococci. However, from day 5 of life, specific diarrhea pathogens (BVR, BCoV, and/or *Cr. parvum*) were detected in all calves with diarrhea (Table 1). It appears that from this point on, diarrhea is caused less by microbial dysbiosis than by specific pathogens. Surprisingly, we could not find any evidence for the presence of *E. coli* F5, a known pathogen in 2–3-day-old diarrheic calves. In contrast to Ewers et al. (2004) who tested individual isolates of *E. coli*, we used, in accordance with the manufacturer's instructions, feces as the test material, which may impair the sensitivity of the test system. In addition, it seems that the incidence of *E. coli* F5 in calves with diarrhea has decreased in recent years (Kolenda et al., 2015).

In conclusion, a restricted development of lactobacilli in the first days of life leads to a quantitative shift in the fecal microbiota. This reflects an enteric dysbiosis that favors the onset of diarrhea in newborn calves. Specific pathogens as causative agents of diarrhea increase over the course of the first week of life. The number of lactobacilli is reduced about 24 h before the clinical manifestation of diarrhea on day 2 after birth. This fact suggests that the incidence of diarrhea in the first days of life might be reduced by administering lactobacilli (especially *L. reuteri*) as early as possible together with colostrum. This hypothesis must be verified using a targeted feeding experiment in an extensive follow-up study.

## Data availability statement

The raw data supporting the conclusions of this article will be made available by the authors, without undue reservation.

## Author contributions

KS: project coordination and support. JS: project implementation. CB: statistics. JB: project leader. All authors contributed to the article and approved the submitted version.

## Conflict of interest

The authors declare that the research was conducted in the absence of any commercial or financial relationships that could be construed as a potential conflict of interest.

## Publisher's note

All claims expressed in this article are solely those of the authors and do not necessarily represent those of their affiliated organizations, or those of the publisher, the editors and the reviewers. Any product that may be evaluated in this article, or claim that may be made by its manufacturer, is not guaranteed or endorsed by the publisher.

## References

[B1] Alak J. I. Wolf B. W. Mdurvwa E. G. Pimentel-Smith G. E. Adeyemo O. (1997). Effect of *Lactobacillus reuteri* on intestinal resistance to *Cryptosporidium parvum* infection in a murine model of acquired immunodeficiency syndrome. J. Infect. Dis. 175, 218–221. 10.1093/infdis/175.1.218 8985225

[B2] Ang L. Y. Too H. K. Tan E. L. Chow T. K. Shek L. P. Tham E. H.. (2016). Antiviral activity of *Lactobacillus reuteri* Protectis against Coxsackievirus A and Enterovirus 71 infection in human skeletal muscle and colon cell lines. Virol. J. 24, 111. 10.1186/s12985-016-0567-6 27341804 PMC4920999

[B3] Arroyo L. G. Rossi L. Santos B. P. Gomez D. E. Surette M. G. Costa M. C. (2020). Luminal and mucosal microbiota of the cecum and large colon of healthy and diarrheic horses. Animals (Basel). 10, 1403. 10.3390/ani10081403 32806591 PMC7460328

[B4] Azad M. A. K. Sarker M. Li T. Yin J. (2018). Probiotic species in the modulation of gut microbiota: an overview. Biomed. Res. Int. 8, 9478630. 10.1155/2018/9478630 29854813 PMC5964481

[B5] Bejuk D. Begovac J. Gamberger D. Kucisec-Tepes N. (2000). Evaluation of phenotypic characteristics for differentiation of enterococcal species using an example based algorithm. Diagn. Microbiol. Infect. Dis. 38, 201–205. 10.1016/s0732-8893(00)00206-6 11146244

[B6] Bendali F. Bichet H. Schelcher F. Sanaa M. (1999). Pattern of diarrhoea in newborn beef calves in south-west France. Vet. Res. 30, 61–74. 10081113

[B7] Bin P. Tang Z. Liu S. Chen S. Xia Y. Liu J.. (2018). Intestinal microbiota mediates enterotoxigenic *Escherichia coli*-induced diarrhea in piglets. BMC Vet. Res. 14, 385. 10.1186/s12917-018-1704-9 30518356 PMC6282381

[B8] Brickell J. S. McGowan M. M. Pfeiffer D. U. Wathes D. C. (2009). Mortality in Holstein-Friesian calves and replacement heifers, in relation to body weight and IGF-I concentration, on 19 farms in England. Animal. 3, 1175–1182. 10.1017/S175173110900456X 22444847

[B9] Cho Y. Yoon K. -J. (2014). An overview of calf diarrhea—infectious etiology, diagnosis, and intervention. J. Vet. Sci. 15, 1–17. 10.4142/jvs.2014.15.1.1 24378583 PMC3973752

[B10] Claß D. F. (1793). Der Hausvater als sein eigener Vieharzt oder wie ein jeder Landwirth sein Rind- Pferd- Schwein- Schaf- und Federvieh selbst und ohne Zuthun anderer von den gewöhnlichsten Krankheiten heilen könne. Leipzig: Christian Gottlieb Hertel Verlag, 61.

[B11] Doll K. Weirather P. Küchle H. (1995). Kälberdurchfall als Bestandsproblem: Betriebsinterne Faktoren und häufige Behandlungsfehler. Der praktische Tierarzt. 1995, 995–1004.

[B12] Donovan G. A. Dohoo I. R. Montgomery D. M. Bennett F. L. (1998). Associations between passive immunity and morbidity and mortality in dairy heifers in Florida, USA. Prev. Vet. Med. 34, 31–46. 9541949 10.1016/S0167-5877(97)00060-3 PMC7134088

[B13] Elze K. Scharfe S. Oppermann C. Gruhle J. Herzog E. (1994). Herdendiagnostische Aspekte bei der neonatalen Kälberdiarrhoe in einer 400er Milchviehanlage. Der praktische Tierarzt. 48–56.

[B14] Ewers C. Schüffner C. Weiss R. Baljer G. Wieler L. H. (2004). Molecular characteristics of *Escherichia coli* serogroup O78 strains isolated from diarrheal cases in bovines urge further investigations on their zoonotic potential. Mol. Nutr. Food Res. 48, 504–514. 10.1002/mnfr.200400063 15538707

[B15] Fallon R. J. Harte F. J. (1983). The occurrence of diarrhoea in calves under different management systems. Ann. Rech. Vet. 14, 473–478. 6677184

[B16] Faul F. Erdfelder E. Lang A. -G. Buchner A. (2007). G^*^Power 3: A flexible statistical power analysis program for the social, behavioral, and biomedical sciences. Behav. Res. Methods 39, 175–191. 10.3758/bf03193146 17695343

[B17] Fischer A. J. Song Y. He Z. Haines D. M. Guan L. L. Steele M. A. (2018). Effect of delaying colostrum feeding on passive transfer and intestinal bacterial colonization in neonatal male Holstein calves. J. Dairy Sci. 101, 3099–3109. 10.3168/jds.2017-13397 29397179

[B18] Gänzle M. G. (2004). Reutericyclin: biological activity, mode of action, and potential applications. Appl. Microbiol. Biotechnol. 64, 326–332. 10.1007/s00253-003-1536-8 14735324

[B19] Gedek B. (1974). Möglichkeiten und Grenzen der mikrobiologischen Futtermittelkontrolle. Dtsch. Tierärztl. Wochenschr. 81, 37–40, 65–69. 4594487

[B20] Girnus D. (2004). Inzidenz und Verlauf von Neugeborenendurchfall bei Kälbern in einem Praxisgebiet in Oberbayern (Ph.D Thesis). Ludwig-Maximilians-University, Munich. 10.52282/edoc.2582

[B21] Glass M. D. Courtney P. D. LeJeune J. T. Ward L. A. (2004). Effects of *Lactobacillus acidophilus* and *lactobacillus reuteri* cell-free supernatants on *Cryptosporidium* viability and infectivity in vitro. Food Microbiol. 21, 423–429. 10.1016/j.fm.2003.11.001

[B22] Gomez D. E. Arroyo L. G. Costa M. C. Viel L. Weese J. S. (2017). Characterization of the fecal bacterial microbiota of healthy and diarrheic dairy calves. J. Vet. Intern. Med. 31, 928–939. 10.1111/jvim.14695 28390070 PMC5435056

[B23] Guard B. C. Barr J. W. Reddivari L. Klemashevich C. Jayaraman A. Steiner J. M.. (2015). Characterization of microbial dysbiosis and metabolomic changes in dogs with acute diarrhea. PLoS ONE. 22, e0127259. 10.1371/journal.pone.0127259 26000959 PMC4441376

[B24] Hartemink R. Rombouts F. M. (1999). Comparison of media for the detection of bifidobacteria, lactobacilli and total anaerobes from faeal samples. J. Microbiol. Methods. 36, 181–192. 10.1016/s0167-7012(99)00031-7 10379804

[B25] Hernández-González J. C. Martínez-Tapia A. Lazcano-Hernández G. García-Pérez B. E. Castrejón-Jiménez N. S. (2021). Bacteriocins from lactic acid bacteria. A powerful alternative as antimicrobials, probiotics, and immunomodulators in veterinary medicine. Animals (Basel). 11, 979. 10.3390/ani11040979 33915717 PMC8067144

[B26] Kaske M. Kunz H. (2003). Handbuch Durchfallerkrankungen der Kälber. Osnabrück: Kamlage Verlag

[B27] Key F. M. Posth C. Esquivel-Gomez L. R. Hübler R. Spyrou M. A. Neumann G. U.. (2020). Emergence of human-adapted *Salmonella enterica* is linked to the Neolithization process. Nat. Ecol. Evol. 4, 324–333. 10.1038/s41559-020-1106-9 32094538 PMC7186082

[B28] Klein-Jöbstl D. Iwersen M. Drillich M. (2014). Farm characteristics and calf management practices on dairy farms with and without diarrhea: a case-control study to investigate risk factors for calf diarrhea. J. Dairy Sci. 97, 5110–5119. 10.3168/jds.2013-7695 24881793 PMC7094405

[B29] Kolenda R. Burdukiewicz M. Schierack P. (2015). A systematic review and meta-analysis of the epidemiology of pathogenic *Escherichia coli* of calves and the role of calves as reservoirs for human pathogenic *E. coli*. Front. Cell Infect. Microbiol. 5, 23. 10.3389/fcimb.2015.00023 25815276 PMC4357325

[B30] Lee S. H. Kim H. Y. Choi E. W. Kim D. (2019). Causative agents and epidemiology of diarrhea in Korean native calves. J. Vet. Sci. 20, e64. 10.4142/jvs.2019.20.e64 31775191 PMC6883198

[B31] Mayer M. Abenthum A. Matthes J. M. Kleeberger D. Ege M. J. Hölzel C.. (2012). Development and genetic influence of the rectal bacterial flora of newborn calves. Vet Microbiol. 161, 179–185. 10.1016/j.vetmic.2012.07.023 22858232

[B32] Millette M. Luquet F. M. Lacroix M. (2007). In vitro growth control of selected pathogens by *Lactobacillus acidophilus*- and *Lactobacillus casei*-fermented milk. Lett. Appl. Microbiol. 44, 314–319. 10.1111/j.1472-765X.2006.02060.x 17309510

[B33] Mokoena M. P. (2017). Lactic acid bacteria and their bacteriocins: classification, biosynthesis and applications against uropathogens: a mini-review. Molecules 22, 1255. 10.3390/molecules22081255 28933759 PMC6152299

[B34] Pace F. Pace M. Quartarone G. (2015). Probiotics in digestive diseases: focus on *Lactobacillus* GG. Minerva Gastroenterol. Dietol. 61, 273–292. 26657927

[B35] Reski-Weide B. (2013). Inzidenz der Neugeborenendiarrhoe bei Kälbern in Abhängigkeit von exogenen Faktoreneine Praxisstudie (Ph.D Thesis). Ludwig-Maximilians-University Munich. 10.5282/edoc.15421

[B36] Rommel M. (2000). Protozoeninfektionen der Wiederkäuer. In: Veterinärmedizinische Parasitologie, eds. M. Rommel, J. Eckert, E. Kutzer, W. Körting, and T. Schnieder (Berlin: Parey Buchverlag), 121–191.

[B37] Schwaiger K. Storch J. Bauer C. Bauer J. (2020). Development of selected bacterial groups of the rectal microbiota of healthy calves during the first week postpartum. Appl. Microbiol. 128, 366–375. 10.1111/jam.14484 31617292 PMC7166559

[B38] Seo B. J. Mun M. R. J R. K Kim C. J. Lee I.. (2010). Bile tolerant *Lactobacillus reuteri* isolated from pig feces inhibits enteric bacterial pathogens and porcine rotavirus. Vet. Res. Commun. 34, 323–333. 10.1007/s11259-010-9357-6 20396947

[B39] Shin D. Chang S. Y. Bogere P. Won K. Choi J. Y. Choi Y. J.. (2019). Beneficial roles of probiotics on the modulation of gut microbiota and immune response in pigs. PLoS ONE. 14, e0220843. 10.1371/journal.pone.0220843 31461453 PMC6713323

[B40] Slanzon G. S. Ridenhour B. J. Moore D. A. Sischo W. M. Parrish L. M. Trombetta S. C.. (2022). Fecal microbiome profiles of neonatal dairy calves with varying severities of gastrointestinal disease. PLoS ONE. 17, e0262317. 10.1371/journal.pone.0262317 34982792 PMC8726473

[B41] Smith H. W. (1965). The development of the flora of the alimentary tract in young animals. J Pathol. Bacteriol. 90, 495–513. 4285022

[B42] Stöber M. (2012). “Kennzeichen, Anamnese, Grundregeln der Untersuchungstechnik, Allgemeine Untersuchung,” in Die klinische Untersuchung des Rindes, eds G. Dirksen, H. -D. Grunder, M. Stöber, Stuttgart (Erlangen: Enke Verlag), 75–141.

[B43] Suchodolski J. S. Foster M. L. Sohail M. U. Leutenegger C. Queen E. V. Steiner J. M.. (2015). The fecal microbiome in cats with diarrhea. PLoS ONE. 10, e0127378. 10.1371/journal.pone.0127378 25992741 PMC4437779

[B44] Talarico T. L. Casas I. A. Chung T. C. Dobrogosz W. J. (1988). Production and isolation of reuterin, a growth inhibitor produced by *Lactobacillus reuteri*. Antimicrob. Agents Chemother. 32, 1854–1858. 10.1128/AAC.32.12.1854 3245697 PMC176032

[B45] Urie N. J. Lombard J. E. Shivley C. B. Kopral C. A. Adams A. E. Earleywine T. J.. (2018). Preweaned heifer management on US dairy operations: Part V. Factors associated with morbidity and mortality in preweaned dairy heifer calves. J. Dairy Sci. 101, 9229–9244. 10.3168/jds.2017-14019 29935825 PMC7094390

[B46] Vieco-Saiz N. Belguesmia Y. Raspoet R. Auclair E. Gancel F. Kempf I.. (2019). Benefits and inputs from lactic acid bacteria and their bacteriocins as alternatives to antibiotic growth promoters during food-animal production. Front. Microbiol. 10, 57. 10.3389/fmicb.2019.00057 30804896 PMC6378274

[B47] Weese J. S. Jelinski M. (2017). Assessment of the fecal microbiota in beef calves. J. Vet. Intern. Med. 31, 176–185. 10.1111/jvim.14611 27873352 PMC5259625

[B48] Youmans B. P. Ajami N. J. Jiang Z. D. Campbell F. Wadsworth W. D. Petrosino J. F.. (2015). Characterization of the human gut microbiome during travelers' diarrhea. Gut Microbes. 6, 110–119. 10.1080/19490976.2015.1019693 25695334 PMC4615231

[B49] Zeineldin M. Aldridge B. Lowe J. (2018). Dysbiosis of the fecal microbiota in feedlot cattle with haemorrhagic diarrhea. Microb. Pathog. 115, 123–130. 10.1016/j.micpath.2017.12.059 29275129

[B50] Zoetendal E. G. Mackie R. I. (2005). “Molecular methods in microbial ecology,” in Probiotics and Prebiotics: Scientific Aspects, ed E. Tannock (Norfolk: Caister Academic Press), 1–24.

