# Supplementary material for: Abundance of selected bacterial groups in healthy calves and calves developing diarrhea during the first week of life: Are there differences before the manifestation of clinical symptoms?
Source: Front Microbiol. 2022 Oct 25;13:958080. doi: 10.3389/fmicb.2022.958080 (PMC9641020; doi:10.3389/fmicb.2022.958080)
Supplement: Supplementary file 1 [file Table_1.DOCX]

Supplement Table: Individual listing of calves (n = 150) with assignment to farm, occurrence of diarrhea, pathogen (detected by ELISA), and colostrum intake (time and volume)

| **Farm** | **Calf** | **Diarrhea within the first week of life (yes/no)** | | **Pathogen detected**  **by ELISA (BIO K 348)** | | | **Colostrum intake** | |
| --- | --- | --- | --- | --- | --- | --- | --- | --- |
| **Number** | **Number** | **yes/no** | **Onset on day** | **BRV** | **BCoV** | ***Cr*** | **Time after birth [h]** | **Volume [L]** |
| 1 | **1_1** | no | - | n.d. | n.d. | n.d. | n.d. | n.d. |
| 1 | **1_2** | yes | 1 | 0 | 0 | 0 | 2 | n.d. |
| 1 | **1_3** | no | - | n.d. | n.d. | n.d. | 1 | 1,5 |
| 1 | **1_4** | yes | 2 | 0 | 0 | 0 | 2 | 0,5 |
| 1 | **1_6** | no | - | n.d. | n.d. | n.d. | 1 | 0,5 |
| 1 | **1_7** | yes | 6 | 0 | 0 | 1 | 1,5 | 1,5 |
| 1 | **1_8** | no | - | n.d. | n.d. | n.d. | 0,5 | 2,5 |
| 1 | **1_9** | no | - | n.d. | n.d. | n.d. | 0,5 | 0,5 |
| 2 | **2_4** | no | - | n.d. | n.d. | n.d. | 0 | 2 |
| 2 | **2_5** | no | - | n.d. | n.d. | n.d. | 0 | 2 |
| 2 | **2_6** | yes | 7 | 1 | 0 | 1 | 0 | 2 |
| 2 | **2_7** | yes | 8 | n.d. | n.d. | n.d. | 2 | 2 |
| 2 | **2_8** | no | - | n.d. | n.d. | n.d. | 0 | 2 |
| 2 | **2_9** | no | - | n.d. | n.d. | n.d. | 0 | 2 |
| 3 | **3_1** | no | - | n.d. | n.d. | n.d. | 7 | 2 |
| 3 | **3_2** | no | - | n.d. | n.d. | n.d. | 0 | 2 |
| 3 | **3_3** | no | - | n.d. | n.d. | n.d. | 3 | 3 |
| 3 | **3_4** | yes | 8 | n.d. | n.d. | n.d. | 0 | 3 |
| 3 | **3_5** | no | - | n.d. | n.d. | n.d. | 3 | 3 |
| 3 | **3_6** | no | - | n.d. | n.d. | n.d. | 3 | 2 |
| 3 | **3_7** | no | - | n.d. | n.d. | n.d. | 0 | 2 |
| 3 | **3_8** | yes | 8 | n.d. | n.d. | n.d. | 0,5 | 2 |
| 3 | **3_9** | yes | 2 | 0 | 0 | 0 | 0,5 | 1,5 |
| 3 | **3_10** | yes | 3 | 0 | 0 | 1 | 3 | 2 |
| 3 | **3_11** | yes | 1 | 0 | 0 | 0 | 3 | 2 |
| 3 | **3_12** | no | - | n.d. | n.d. | n.d. | 12 | 3 |
| 3 | **3_13** | no | - | n.d. | n.d. | n.d. | 1 | 1 |
| 4 | **4_2** | no | - | n.d. | n.d. | n.d. | 0,5 | 1,5 |
| 4 | **4_3** | no | - | n.d. | n.d. | n.d. | 2 | 1,5 |
| 4 | **4_4** | no | - | n.d. | n.d. | n.d. | 2 | 2 |
| 4 | **4_6** | yes | 6 | 0 | 0 | 1 | 2 | 2 |
| 4 | **4_7** | no | - | n.d. | n.d. | n.d. | 0,5 | 1,5 |
| 4 | **4_8** | yes | 2 | 0 | 0 | 1 | 0,5 | 1,5 |
| 4 | **4_9** | yes | 2 | 0 | 0 | 1 | 0 | 1,5 |
| 4 | **4_10** | no | - | n.d. | n.d. | n.d. | 3 | 1 |
| 4 | **4_11** | no | - | n.d. | n.d. | n.d. | 0,5 | 1 |
| 4 | **4_12** | no | - | n.d. | n.d. | n.d. | 1,5 | 1,5 |
| 4 | **4_13** | yes | 8 | n.d. | n.d. | n.d. | 0,5 | 1,5 |
| 4 | **4_14** | yes | 1 | 0 | 0 | 0 | 0,5 | 1,5 |
| 4 | **4_15** | yes | 6 | 0 | 0 | 1 | 0,5 | 1 |
| 5 | **5_1** | yes | 7 | 0 | 0 | 1 | 0 | 1,5 |
| 5 | **5_2** | yes | 8 | n.d. | n.d. | n.d. | 0 | 1 |
| 5 | **5_3** | no | - | n.d. | n.d. | n.d. | 0,5 | 1 |
| 5 | **5_5** | yes | 2 | 0 | 0 | 1 | 12 | 0,5 |
| 5 | **5_6** | yes | 6 | 0 | 0 | 1 | 0 | 1,5 |
| 5 | **5_7** | yes | 1 | 0 | 0 | 0 | 0 | 2 |
| 5 | **5_9** | yes | 2 | 0 | 0 | 0 | 1 | 1,5 |
| 5 | **5_10** | yes | 5 | 1 | 0 | 1 | 6 | 1 |
| 5 | **5_11** | yes | 1 | 0 | 0 | 0 | 0 | 0,5 |
| 5 | **5_12** | yes | 2 | 0 | 0 | 0 | 0 | 0,5 |
| 5 | **5_13** | yes | 6 | 1 | 0 | 1 | 2 | 0,5 |
| 5 | **5_14** | yes | 2 | 0 | 0 | 0 | 0 | 1 |
| 5 | **5_15** | yes | 2 | 0 | 0 | 1 | n.d. | 1,5 |
| 6 | **6_1** | yes | 2 | 0 | 0 | 0 | 0 | 1,5 |
| 6 | **6_2** | yes | 2 | 0 | 0 | 1 | 0,5 | 1,5 |
| 6 | **6_3** | yes | 6 | 0 | 0 | 1 | 0,5 | 1,5 |
| 6 | **6_4** | no | - | n.d. | n.d. | n.d. | 1 | 3 |
| 6 | **6_5** | no | - | n.d. | n.d. | n.d. | 0 | 2 |
| 6 | **6_6** | no | - | n.d. | n.d. | n.d. | 0 | 3 |
| 6 | **6_7** | no | - | n.d. | n.d. | n.d. | 0 | 2 |
| 6 | **6_8** | yes | 7 | 0 | 0 | 1 | 0 | 3,5 |
| 6 | **6_9** | yes | 1 | 0 | 0 | 1 | 1 | 2 |
| 6 | **6_10** | no | - | n.d. | n.d. | n.d. | 0 | 2 |
| 6 | **6_11** | yes | 6 | 0 | 0 | 1 | 1 | 1,5 |
| 6 | **6_12** | no | - | n.d. | n.d. | n.d. | 0,5 | 2 |
| 6 | **6_13** | yes | 3 | 1 | 0 | 1 | 0,5 | 1,5 |
| 6 | **6_14** | no | - | n.d. | n.d. | n.d. | 0,5 | 2 |
| 6 | **6_15** | no | - | n.d. | n.d. | n.d. | 0,5 | 1,5 |
| 7 | **7_1** | yes | 6 | 1 | 0 | 1 | 6,5 | 1,5 |
| 7 | **7_2** | yes | 6 | 1 | 0 | 1 | 3 | 1,5 |
| 7 | **7_3** | yes | 2 | 0 | 0 | 0 | 2 | 1 |
| 7 | **7_4** | yes | 2 | 0 | 0 | 0 | 3 | 1 |
| 7 | **7_5** | yes | 2 | 0 | 0 | 0 | 3 | 1,5 |
| 7 | **7_6** | no | - | n.d. | n.d. | n.d. | 1 | 2 |
| 7 | **7_7** | no | - | n.d. | n.d. | n.d. | 2 | 2 |
| 7 | **7_8** | no | - | n.d. | n.d. | n.d. | 0,5 | 1 |
| 7 | **7_9** | yes | 7 | 1 | 0 | 0 | 0,5 | 2 |
| 7 | **7_10** | no | - | n.d. | n.d. | n.d. | 2 | 1,5 |
| 7 | **7_15** | yes | 5 | 1 | 0 | 0 | 1 | 2 |
| 7 | **7_18** | yes | 7 | 1 | 0 | 0 | 11 | n.d. |
| 8 | **8_1** | yes | 8 | n.d. | n.d. | n.d. | 0 | 2 |
| 8 | **8_2** | yes | 12 | n.d. | n.d. | n.d. | 4 | 2 |
| 8 | **8_3** | no | - | n.d. | n.d. | n.d. | 0 | 2 |
| 8 | **8_4** | no | - | n.d. | n.d. | n.d. | 0 | 2 |
| 8 | **8_5** | no | - | n.d. | n.d. | n.d. | 0 | 2 |
| 8 | **8_6** | yes | 13 | n.d. | n.d. | n.d. | 0 | 3 |
| 8 | **8_8** | no | - | n.d. | n.d. | n.d. | 0 | 1,5 |
| 8 | **8_9** | yes | 11 | n.d. | n.d. | n.d. | 0,5 | 1,5 |
| 8 | **8_10** | yes | 9 | n.d. | n.d. | n.d. | 2 | 2 |
| 8 | **8_11** | yes | 8 | n.d. | n.d. | n.d. | 0 | 1 |
| 8 | **8_12** | yes | 11 | n.d. | n.d. | n.d. | 0 | 2 |
| 8 | **8_13** | no | - | n.d. | n.d. | n.d. | 0 | 2,5 |
| 8 | **8_14** | no | - | n.d. | n.d. | n.d. | 0 | 4 |
| 8 | **8_15** | no | - | n.d. | n.d. | n.d. | 1 | 2 |
| 9 | **9_1** | yes | 3 | 0 | 0 | 0 | 2 | 1,5 |
| 9 | **9_2** | yes | 4 | 0 | 0 | 1 | 2 | 1,5 |
| 9 | **9_4** | yes | 4 | 0 | 0 | 0 | 3 | 1 |
| 9 | **9_5** | yes | 3 | 0 | 0 | 1 | 5 | 1 |
| 10 | **10_1** | no | - | n.d. | n.d. | n.d. | 4 | 1,5 |
| 10 | **10_2** | no | - | n.d. | n.d. | n.d. | 0,5 | 1,5 |
| 10 | **10_3** | no | - | n.d. | n.d. | n.d. | 1,5 | 1,5 |
| 10 | **10_4** | no | - | n.d. | n.d. | n.d. | 0,5 | 1,5 |
| 10 | **10_6** | no | - | n.d. | n.d. | n.d. | 11 | 1,5 |
| 10 | **10_7** | yes | 5 | 0 | 1 | 0 | 1 | 1,5 |
| 10 | **10_8** | no | - | n.d. | n.d. | n.d. | 1,5 | 1,5 |
| 10 | **10_9** | no | - | n.d. | n.d. | n.d. | 1 | 1,5 |
| 10 | **10_10** | no | - | n.d. | n.d. | n.d. | 0,5 | 2 |
| 10 | **10_11** | no | - | n.d. | n.d. | n.d. | 2 | 1 |
| 10 | **10_12** | no | - | n.d. | n.d. | n.d. | 1 | 1,5 |
| 10 | **10_13** | no | - | n.d. | n.d. | n.d. | 1 | 1,5 |
| 10 | **10_14** | no | - | n.d. | n.d. | n.d. | 1 | 2 |
| 10 | **10_15** | no | - | n.d. | n.d. | n.d. | 1 | 1,5 |
| 11 | **11_1** | yes | 6 | 1 | 0 | 0 | 0,5 | 1 |
| 11 | **11_2** | yes | 5 | 1 | 0 | 0 | 3 | 2 |
| 11 | **11_3** | no | - | n.d. | n.d. | n.d. | 6,5 | 2 |
| 11 | **11_4** | no | - | n.d. | n.d. | n.d. | 0,5 | 1 |
| 11 | **11_5** | no | - | n.d. | n.d. | n.d. | 0,5 | 1 |
| 11 | **11_6** | yes | 6 | 1 | 0 | 0 | 2 | 3 |
| 11 | **11_7** | no | - | n.d. | n.d. | n.d. | 3,5 | 3 |
| 11 | **11_8** | no | - | n.d. | n.d. | n.d. | 1,5 | 3 |
| 11 | **11_9** | yes | 6 | 1 | 0 | 0 | 0,5 | 3 |
| 11 | **11_10** | yes | 6 | 1 | 0 | 0 | 1 | 2 |
| 11 | **11_11** | yes | 6 | 1 | 0 | 0 | 2 | 2 |
| 11 | **11_12** | no | - | n.d. | n.d. | n.d. | 1 | 3 |
| 11 | **11_13** | no | - | n.d. | n.d. | n.d. | 2 | 3 |
| 11 | **11_14** | no | - | n.d. | n.d. | n.d. | 0,5 | 1 |
| 11 | **11_15** | yes | 4 | 1 | 0 | 0 | 0,5 | 0,5 |
| 11 | **11_16** | no | - | n.d. | n.d. | n.d. | 2 | 2 |
| 11 | **11_17** | no | - | n.d. | n.d. | n.d. | 7 | 3 |
| 11 | **11_18** | yes | 8 | n.d. | n.d. | n.d. | 5 | 2,5 |
| 12 | **12_1** | yes | 2 | 0 | 1 | 0 | 1,5 | 3,5 |
| 12 | **12_2** | yes | 3 | 0 | 1 | 0 | 2,5 | 1 |
| 12 | **12_3** | yes | 5 | 0 | 1 | 0 | 2,5 | 3 |
| 12 | **12_4** | yes | 7 | 0 | 1 | 0 | 2 | 2 |
| 12 | **12_5** | yes | 7 | 0 | 1 | 0 | 2,5 | 2 |
| 12 | **12_6** | no | - | n.d. | n.d. | n.d. | 3 | 3 |
| 12 | **12_7** | yes | 7 | 0 | 1 | 0 | 3 | 1 |
| 12 | **12_8** | no | - | n.d. | n.d. | n.d. | 1 | 3,5 |
| 13 | **13_1** | yes | 2 | 0 | 0 | 0 | n.d. | n.d. |
| 13 | **13_2** | no | - | n.d. | n.d. | n.d. | n.d. | n.d. |
| 13 | **13_3** | no | - | n.d. | n.d. | n.d. | n.d. | n.d. |
| 13 | **13_4** | no | - | n.d. | n.d. | n.d. | n.d. | n.d. |
| 13 | **13_5** | no | - | n.d. | n.d. | n.d. | n.d. | n.d. |
| 13 | **13_6** | no | - | n.d. | n.d. | n.d. | n.d. | n.d. |
| 13 | **13_7** | no | - | n.d. | n.d. | n.d. | n.d. | n.d. |
| 13 | **13_8** | no | - | n.d. | n.d. | n.d. | n.d. | n.d. |
| 13 | **13_9** | no | - | n.d. | n.d. | n.d. | n.d. | n.d. |
| 13 | **13_10** | no | - | n.d. | n.d. | n.d. | n.d. | n.d. |
| 13 | **13_11** | no | - | n.d. | n.d. | n.d. | n.d. | n.d. |
| 13 | **13_12** | no | - | n.d. | n.d. | n.d. | n.d. | n.d. |

BRV: bovine rotavirus; BCoV: bovine coronavirus; Cr: *Cryptosporidium parvum;* n.d.: no data
